# Supplementary material for: The phylodynamics of SARS-CoV-2 during 2020 in Finland
Source: Commun Med (Lond). 2022 Jun 10;2:65. doi: 10.1038/s43856-022-00130-7 (PMC9187640; doi:10.1038/s43856-022-00130-7)
Supplement: Supplementary file 1 — Description of Additional Supplementary Files [file 43856_2022_130_MOESM1_ESM.pdf]

Description of Additional Supplementary Files

File Name: Supplementary Data 1

Description: Complete list of primer pools targeting SARS-CoV-2 used in the study.

File Name: Supplementary Data 2

Description: GISAID acknowledgements table including accession ID, originating laboratory, submitting laboratory and authors.

File Name: Supplementary Data 3

Description: Primary data for Figures 1, 3 and 4.
