# Supplementary material for: The phylodynamics of SARS-CoV-2 during 2020 in Finland
Source: Commun Med (Lond). 2022 Jun 10;2:65. doi: 10.1038/s43856-022-00130-7 (PMC9187640; doi:10.1038/s43856-022-00130-7)
Supplement: Supplementary file 5 — Supplementary Information [file 43856_2022_130_MOESM5_ESM.pdf]

Supplementary Table 1: Number of unique sequences and cumulative number of cases for 17 European countries, including Finland and 16 selected European countries. Sequences were from samples collected in spring of 2020.

Country	Unique sequences (n)	Cumulative cases (n)	Proportion of sampled sequences (%)	Downsampled sequences (n)
Estonia	4	1,459	0.27	4
Latvia	19	682	3.37	17
Norway	46	6,937	0.66	44
Hungary	47	1,763	3.01	46
Poland	54	8,379	0.7	54
Turkey	62	78,546	0.08	59
Sweden	134	13,743	1.78	100
Netherlands	148	30,619	0.67	100
Austria	187	14,595	1.43	100
Denmark	243	7,268	5.79	100
Finland	340	3,489	13.87	333
Italy	496	172,434	0.34	129
Germany	654	141,397	0.61	104
Switzerland	682	27,078	2.97	100
Spain	847	190,839	0.46	143
France	1,185	147,057	0.88	110
United Kingdom	1,835	117,798	1.95	100
Total	6,983	964,083	35.55	1,643

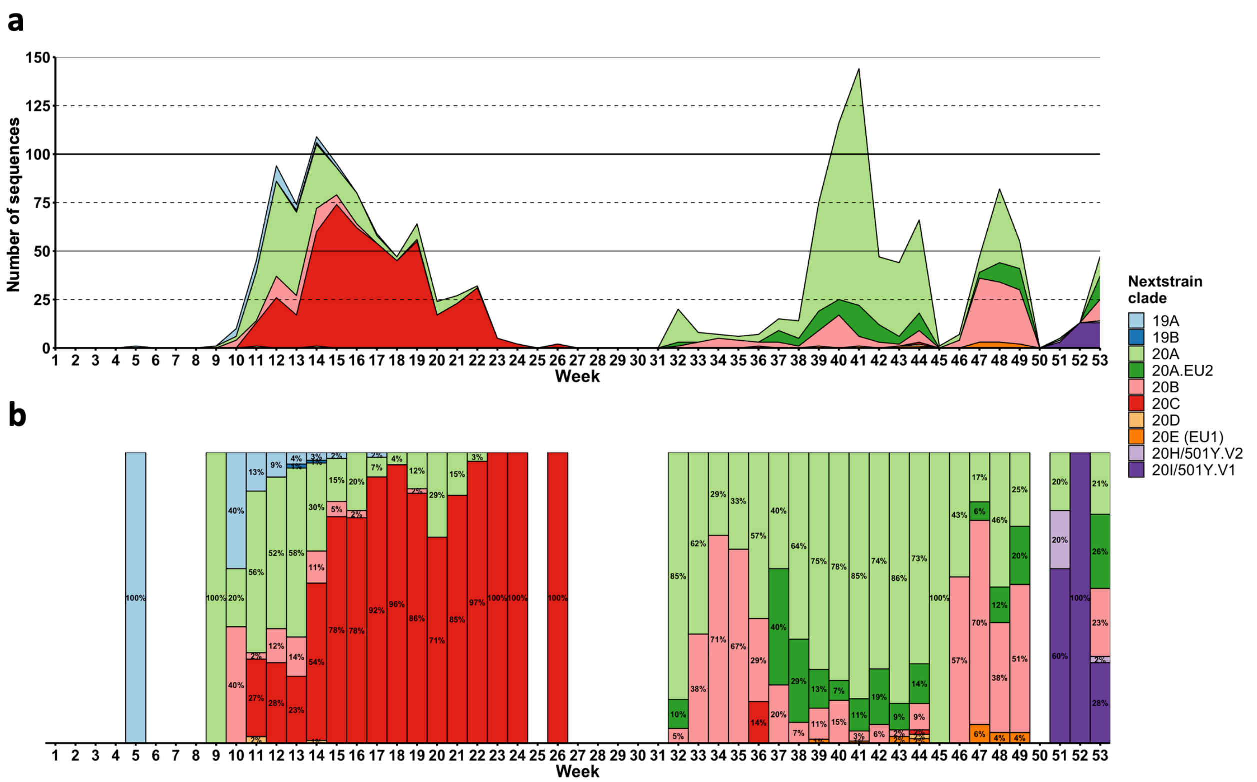

Supplementary Fig. 1: Nexstrain clade distributions of SARS-CoV-2 (n = 1,597) in Finland in 2020. The number of sequences per week are displayed in panel **(A)** and clade proportions are shown in panel **(B)**. The Nextstrain clade 20C stands out as it was the most prominent strain and was only detected during the first pandemic wave (weeks 9–24) in spring in 2020. Clade assignment was done with the Nextclade tool.

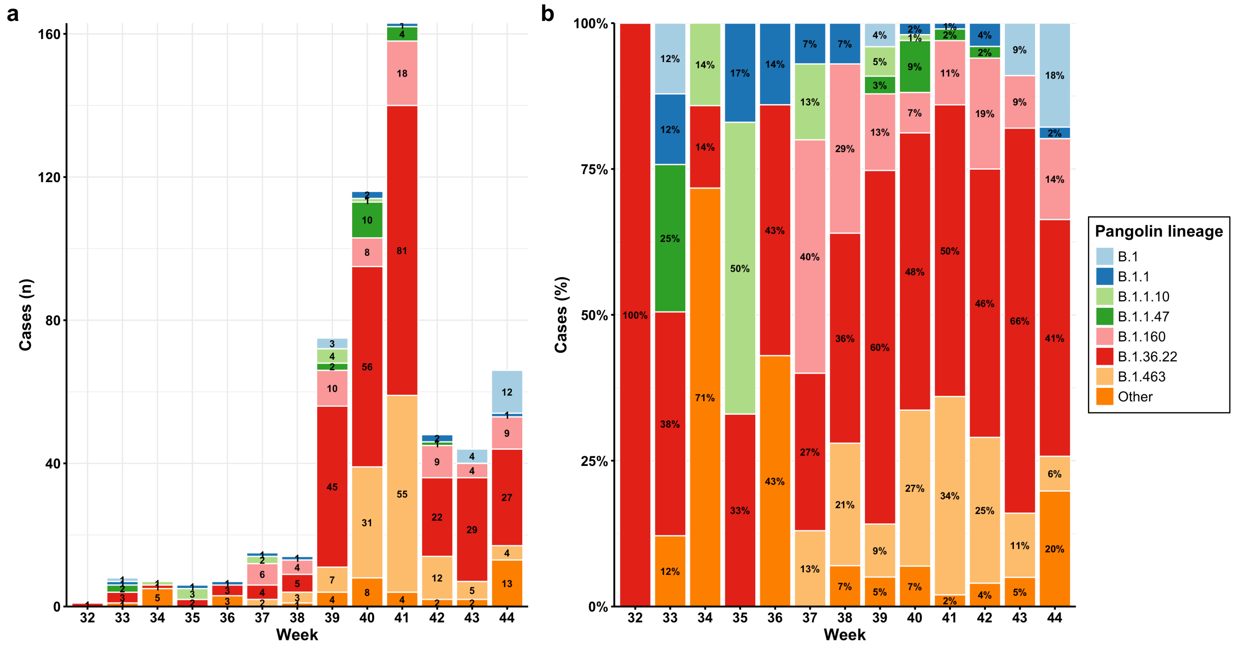

Supplementary Fig. 2: Turnover of Finnish SARS-CoV-2 lineages in fall of 2020 (weeks 32–44). During weeks 32–38 many lineages were detected among Finnish cases with relatively similar frequencies until B.1.36.22 and B.1.463 (both Finland-specific lineages) became the most prevalent lineages beginning in week 39. Panel **(A)** depicts the number of SARS-Cov-2 cases and lineages detected in the HUS area per week and panel **(B)** shows the proportions (%) of lineages per week.

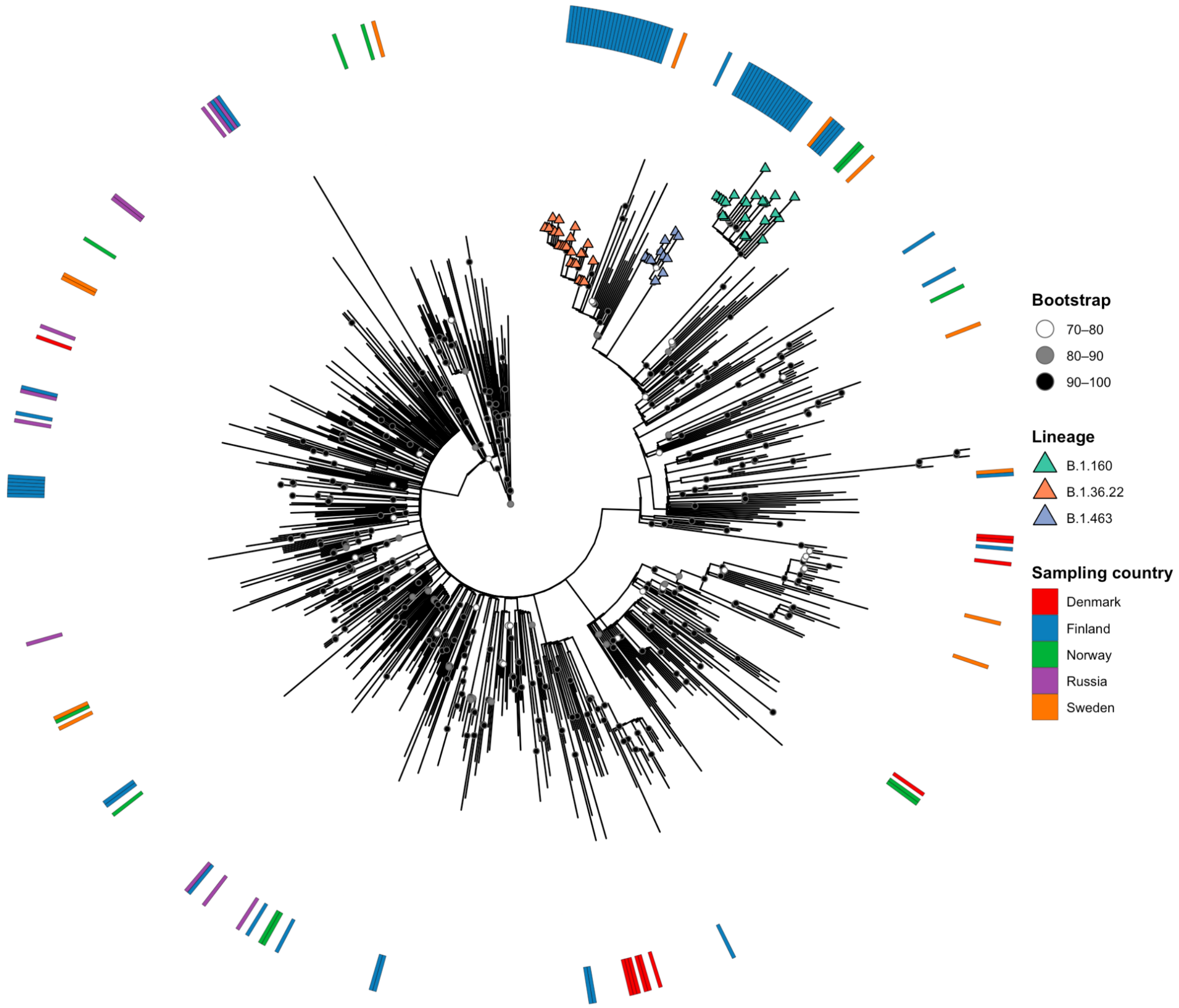

Supplementary Fig. 3: Phylogenetic trees of Finnish SARS-CoV-2 sequences from fall of 2020. The trees were constructed from Finnish sequences (n = 77) and a reference set of SARS-CoV-2 sequences from different countries around the globe (n = 745). Sequences from the neighboring countries of Finland were highlighted in the trees. All sequences were obtained from the GISAID database and include complete genomes with full collection dates from July 15 to September 30, 2020. Finnish sequences were collected between August 8 and September 18, 2020 (weeks 32–38). The nucleotide sequences were aligned with MAFFT and 50 characters were trimmed from the resulting alignment. The maximum-likelihood tree was computed with the COVID-19 version of IQ-TREE with 1,000 bootstraps. The Wuhan reference strain (NC_045512.2) was used as an outgroup and the root.

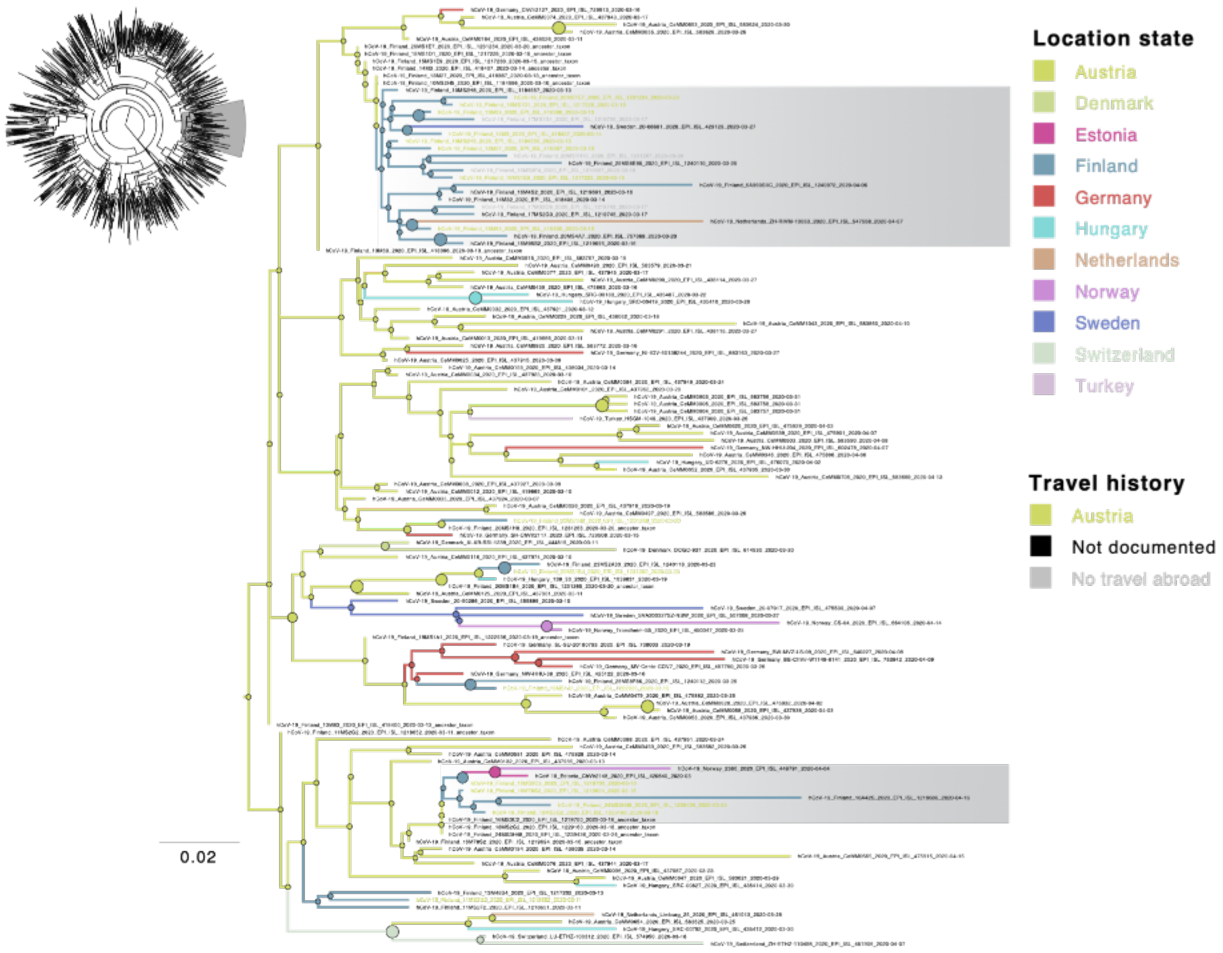

Supplementary Fig. 4: Maximum Clade Credibility (MCC) phylogenetic trees of SARS-CoV-2 sequences as obtained from Bayesian phylogeographic reconstructions including individual travel history information. Inset shows a MCC phylogenetic tree including 1,643 genomes from 17 European selected countries, including Finland. One cluster containing predominantly Austrian genomes is shaded in gray and shown in more detail in the main Figure**.** Main Figure displays an MCC phylogenetic tree with branches and nodes coloured according to the posterior location states, and node circles sized according to the posterior node support. The tip labels are coloured according to travel history information, which is only available for the Finnish SARS-CoV-2 genomes. Two clusters containing predominantly Finnish genomes are shaded in gray.

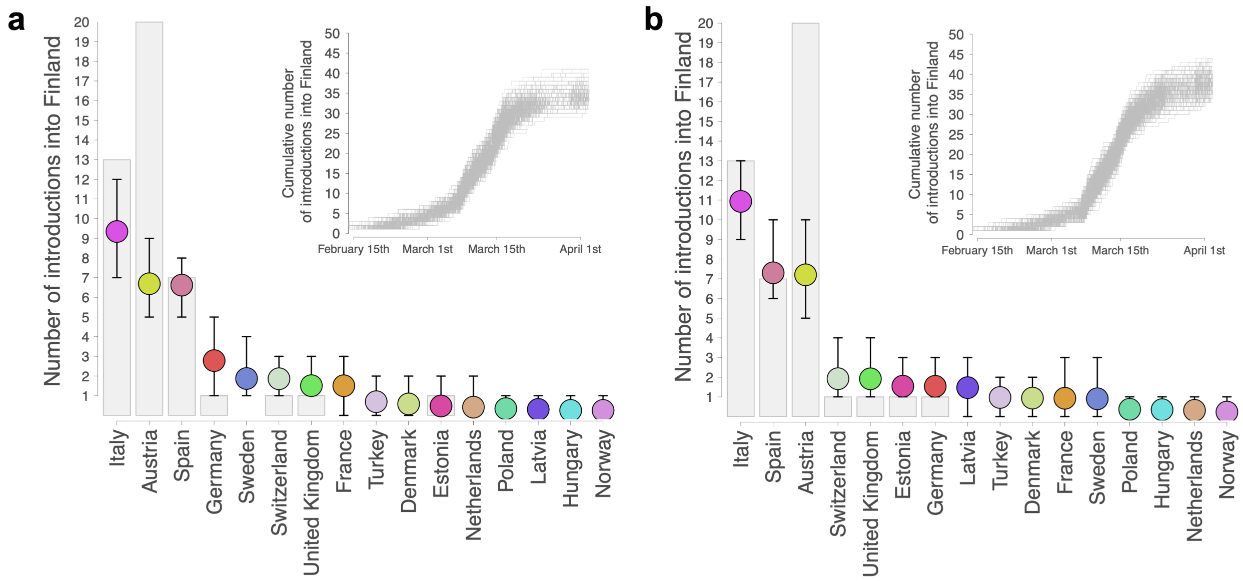

Supplementary Fig. 5: Mean and 95% highest posterior density (HPD) number of transitions to Finland from each of the 16 selected European countries. (A) Discrete phylogeographic reconstructions including unsampled taxa. **(B)** Discrete phylogeographic reconstructions including unsampled taxa and downsampled Finnish taxa. Gray bars indicate the number of cases with travel history data returning from each country. Inset shows the cumulative number of introductions into Finland summarized from a posterior sample of location-annotated phylogenetic trees.

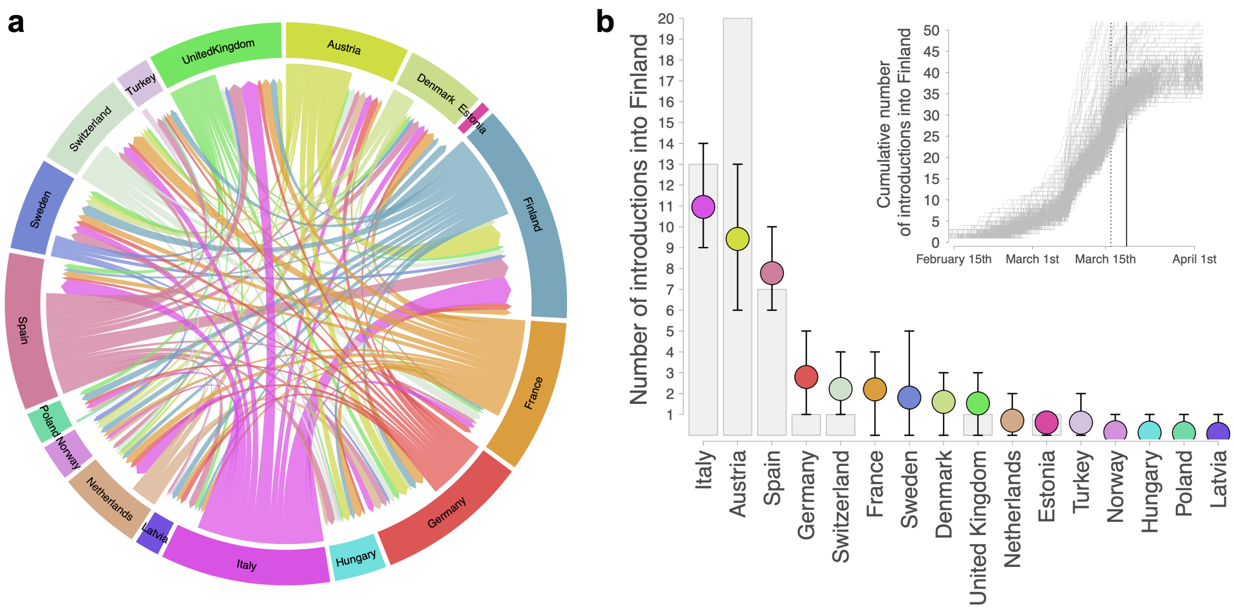

Supplementary Fig. 6: Discrete phylogeographic reconstruction of SARS-CoV-2 introductions into Finland during the first wave epidemic under the Skygrid model. (A) Circular migration flow plot based on the posterior expectations of the Markov jumps between 17 country-level locations, including Finland and 16 selected European countries. Migration flow out of a particular location starts close to the outer ring and ends with an arrowhead more distant from the destination location. **(B)** Mean and 95% highest posterior density (HPD) number of transitions to Finland from each of the 16 selected European countries, as estimated from 1,000 trees subsampled from the posterior distribution. Gray bars indicate the number of cases with travel history data returning from each country. Inset shows the cumulative number of introductions into Finland summarized from a posterior sample of phylogeographic trees. Dashed line indicates the day (16th of March) when the Finnish authorities declared a state of emergency due to COVID-19. Full line indicates the day (19th of March) when the Finnish authorities announced a restriction of passenger traffic at Finland's borders.

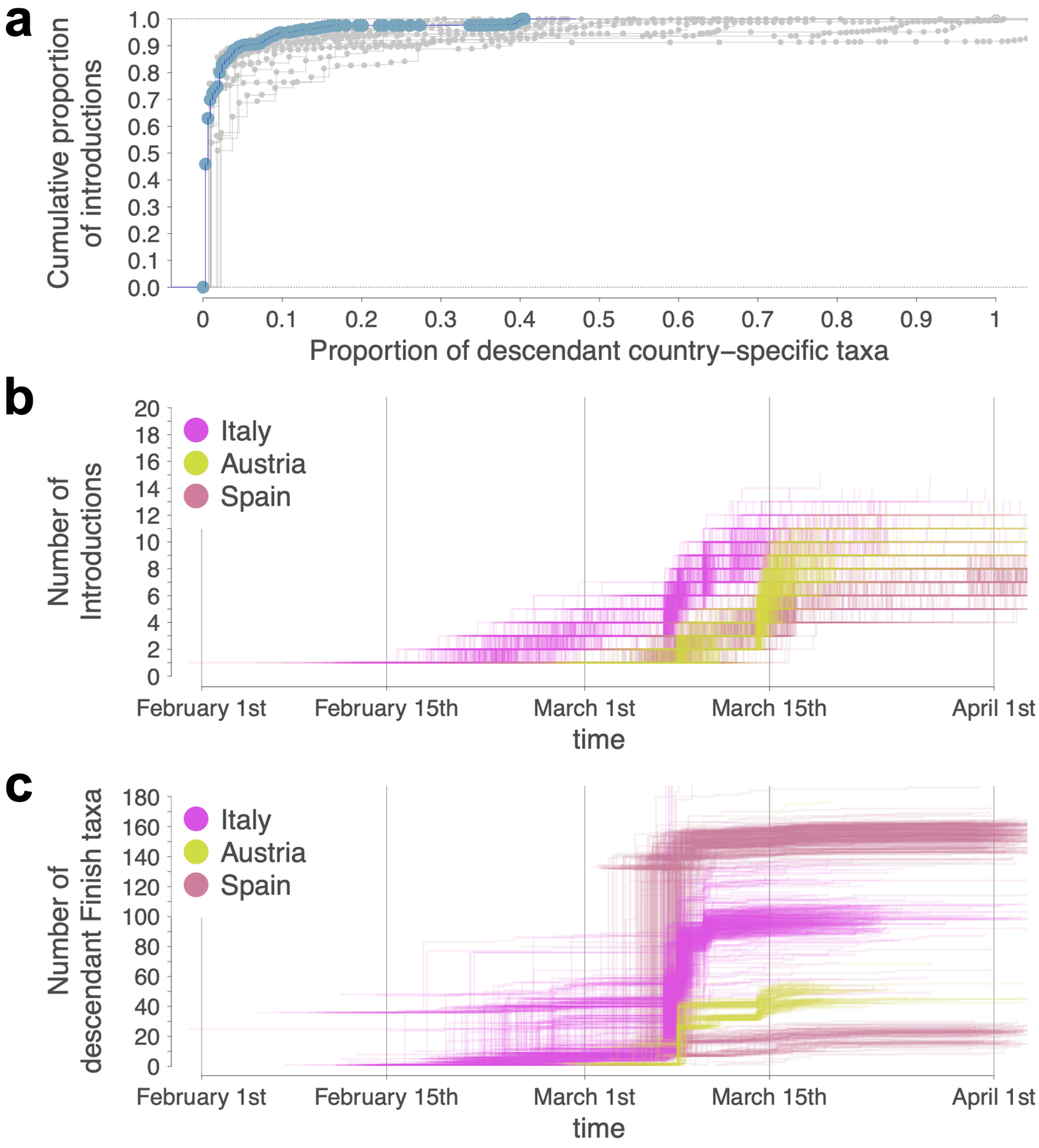

Supplementary Fig. 7: Empirical cumulative distribution function plot and temporal cumulative number of introductions under the Skygrid model. (A) The proportion of descendant country-specific taxa (blue for Finland and gray for each of the 16 selected European countries excluding Latvia and Estonia for which few genomes were available) reflects the proportion of taxa from a given country that are descendants from a given introduction. The figure shows that the majority of introductions are responsible for a relatively small fraction of the taxa sampled in a given country, while a few introductions are responsible for a large proportion of the taxa sampled in a given country. Cumulative number of phylogeographic transitions **(B)** and cumulative number of Finnish descendant state taxa **(C)** over time from Italy, Austria and Spain to Finland.

Supplementary Note 1. Additional information about the introduction and spread of SARS-CoV-2 in Finland during 2020

Two positive cases were initially reported in week 9 (February 25 and 27, 2020) within the Hospital District of Helsinki and Uusimaa (HUS) area during the onset of the SARS-CoV-2 pandemic in Finland. This area covers the capital region (includes Helsinki, Vantaa, Espoo and Kauniainen), which is the most inhabited (ca. 1.2 million residents of the ca. 5.5 million total population) and the most densely populated area (ca. 1,680 residents per km^2 on average) in Finland (Supplementary Fig. 8) [1]. The number of reported cases subsequently nearly tripled from six cases in week 9 to 16 in week 10 (beginning of March) and the number of diagnosed cases reached 196 on March 13, 2020 (week 11). The government declared a national emergency and closed all public schools and government buildings starting March 16, 2020 (week 12) [2].

Despite these responses the number of cases continued to rise. Travel restrictions to and from the Uusimaa region were imposed by the Finnish parliament on March 27, 2020 (week 13), and subsequently lifted on April 15, 2020 (week 16). The number of weekly cases spiked during April (weeks 14–17) with over 600 cases. The number of tests performed increased rapidly from less than 100 per day to over 10,000 per day during this period (weeks 9–17, i.e. from end of February to mid-April). The test positivity rate dropped during the same time from ca. 21.4% (six detections from 28 tests) to 5.6% (611 positive cases from 10,853 tests). As the number of positive SARS-CoV-2 cases started to decrease, kindergartens and elementary schools were reopened on May 14 (week 20). In the beginning of June (week 23), restrictions on social gatherings were partially loosened to allow larger gatherings of 10–50 people in public spaces (e.g., restaurants and sport events). The rate of weekly detections steadily decreased to less than 100 during this time, and the number of tests per week also dropped to half during summer until week 26 (end of June) [3]. International travel restrictions were lifted between European countries with low infection rates (less than 25 per 100,000) [4] on July 8, 2020 (week 28). Weekly SARS-CoV-2 cases in the HUS

area remained low with less than 50 cases per week for seven weeks until week 31 (end of July). The number of weekly tests performed steadily increased over the rest of summer and fall.

The second epidemic wave began in week 32 (beginning of August) with approximately 100 new cases each week until week 37 (beginning of September). After this point, the number of positive cases increased by on average 100 cases per week until peaking at approximately 1,600 cases per week. During this time, the rates of SARS-CoV-2 detections were well below 200 until mid-September (week 38), when the number of cases started to rise.

The peak of the second SARS-CoV-2 wave lasted from week 47 to 50 (from mid-November to the beginning of December). Over 51,000 tests were performed during the peak in week 48 (end of November). Compared to the previous peak in spring, the winter peak had nearly 2.5 times more positive detections. However, the detection rate at the time was 4.0% (1,662 detections from 41,769 tests), lower than in spring peak. Nearing the end of the year, the number of weekly positive findings then sharply dropped to approximately 900, almost half compared to the peak of the second wave.

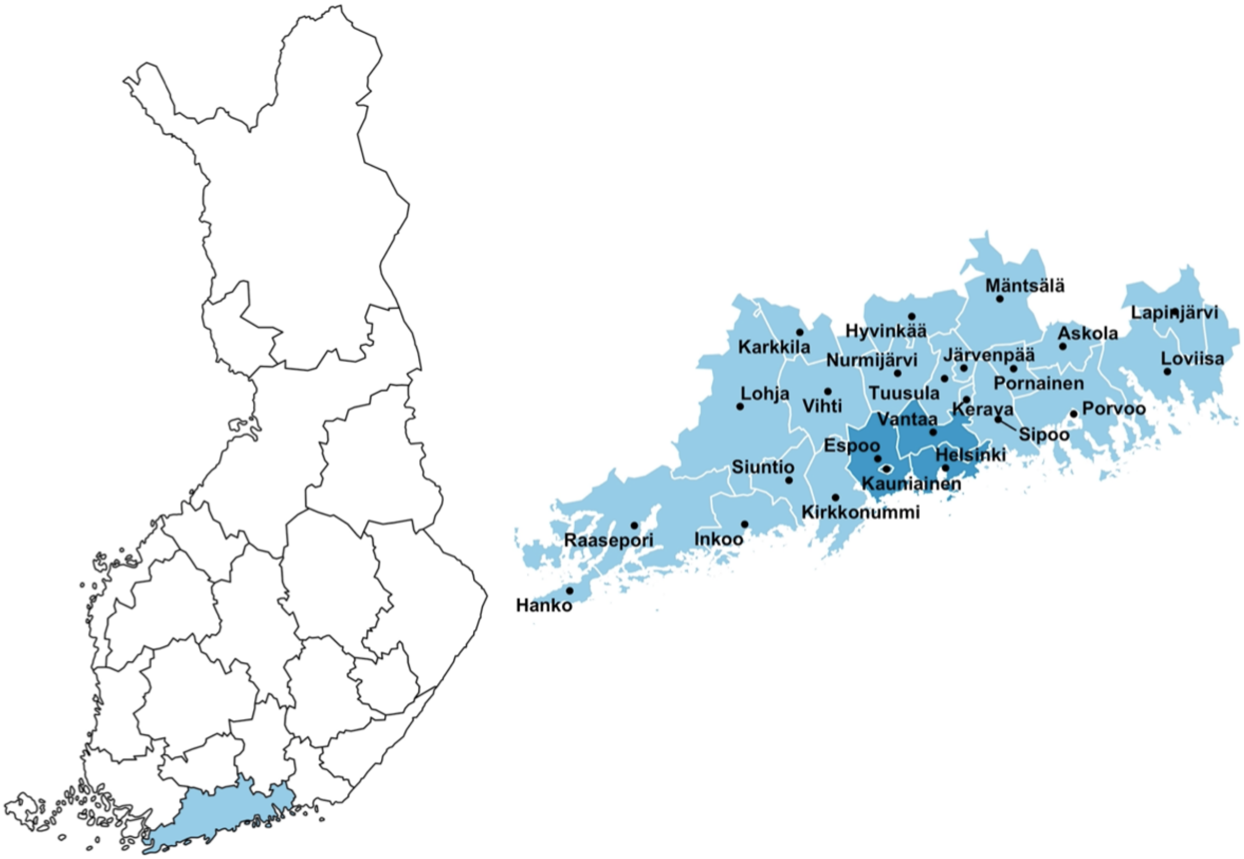

Supplementary Fig. 8: Map of the Hospital District of Helsinki and Uusimaa (HUS) and municipalities belonging to it in Finland. The capital region, which includes the municipalities of Helsinki, Espoo, Vantaa and Kauniainen, is highlighted in darker blue.

Supplementary References

1. Jarva, H. *et al.* Laboratory-based surveillance of COVID-19 in the Greater Helsinki area, Finland, February-June 2020. *Int. J. Infect. Dis.* **104,** 111–116 (2021).
2. Finnish Government. Government, in cooperation with the President of the Republic, declares a state of emergency in Finland over coronavirus outbreak. *Finnish Government* (2020). at <https://valtioneuvosto.fi/-/10616/hallitus-totesi-suomen-olevan-poikkeusoloissa-koronavirustilanteen-vuoksi?languageId=en_US>
3. Willberg, E., Järv, O., Väisänen, T. & Toivonen, T. Escaping from Cities during the COVID-19 Crisis: Using Mobile Phone Data to Trace Mobility in Finland. *ISPRS Int J Geoinf* **10,** 103 (2021).

4. Finnish Institute for Health and Welfare (THL). Traffic light model to help in the assessment of risks associated with foreign travel. at <https://thl.fi/en/web/infectious-diseases-and-vaccinations/what-s-new/coronavirus-covid-19-latest-updates/travel-and-the-coronavirus-pandemic/traffic-light-model-to-help-in-the-assessment-of-risks-associated-with-foreign-travel>
